# Response to written feedback of clinical data within a longitudinal study: a qualitative study exploring the ethical implications

**DOI:** 10.1186/1471-2288-11-10

**Published:** 2011-01-27

**Authors:** Karen Lorimer, Cindy M Gray, Kate Hunt, Sally Wyke, Annie Anderson, Michaela Benzeval

**Affiliations:** 1 MRC/CSO Social and Public Health Sciences Unit, 4 Lilybank Gardens, Glasgow, G12 8RZ, UK; 2 Institute for Applied Health Research, Glasgow Caledonian University, Cowcaddens Road, Glasgow, G4 0BA, UK; 3 Alliance for Self Care Research, University of Stirling, Stirling, FK9 4LA, UK; 4 Centre for Public Nutrition Research, University of Dundee, Dundee, DD1 4HN, UK

## Abstract

**Background:**

There is a growing ethical imperative to feedback research results to participants but there remains a striking lack of empirical research on how people respond to individualised feedback. We sought to explore longitudinal study participants' response to receiving individual written feedback of weight-related and blood results, and to consider the balance of harms against benefits.

**Methods:**

A qualitative study with face-to-face and telephone interviews conducted with 50 men and women who had participated in the fifth and most recent wave of the cohort study 'West of Scotland Twenty-07' and received a feedback letter containing body mass index (BMI), body fat percentage, cholesterol and glycated haemoglobin A_1c _(HbA_1__c_) results.

**Results:**

Expectations of, and response to, the feedback of their individual results varied. Whilst half of the participants were on the whole 'pleased' with their results or held neutral views, half reported negative responses such as 'shock' or 'concern', particularly in relation to the weight-related results. Participants who were overweight and obese used the most negative language about their results, with some being quite distressed and reporting feelings of powerlessness, low self-image and anxiety over future health. Nevertheless, some people reported having implemented lifestyle changes in direct response to the feedback, resulting in significant weight-loss and/or dietary improvements. Others reported being motivated to change their behaviour. Age and gender differences were apparent in these narratives of behaviour change.

**Conclusions:**

The potential harm caused to some participants may be balanced against the benefit to others. More evaluation of the impact of the format, content and means of individualised feedback of research findings in non-trial studies is required given the growing ethical imperative to offer participants a choice of receiving their results, and the likelihood that a high percentage will choose to receive them.

## Background

The issue of returning research results to participants has been receiving increasing attention internationally [1], and the evidence-base on this has grown in the past decade [2]. Largely an ethical debate, the key issue has been whether it is appropriate to deny participants their results [3]. Despite this debate, confusion surrounds the issue of feeding back data to research participants, in part due to: the plethora of national and international ethics guidelines that exist [1]; few exploratory studies that report participants' experiences of receiving feedback at all, with a particular lack of research on participants' views towards receiving individualised feedback [4]; and variation in the reported ethical balance of beneficence (intent to benefit) against nonmalefience (intent not to harm) [5,6]. It is important to continue to examine the potential that feedback has for harm as well as good, so that any feedback given is ethically robust and avoids becoming a simple box-ticking exercise.

There has been a 'mushrooming' of interest - and indeed policy within the United Kingdom (UK) context [7,8] - around whether and how to feed back data to participants of clinical trials. The Medical Research Council (MRC) in the UK, for example, has produced guidelines on the use of human tissue and biological samples, which recommended that research participants have the right 'to know individual research results that affect their interests', adding they 'should be able to choose whether to exercise that right.' [7] Despite the increasing interest, there remains a stark paucity of evidence on participants' responses to receiving feedback. A recent (non-systematic) review found eight studies which empirically examined the response to feedback, but seven concerned aggregate results and no community-based study was included [4]. An editorial in the *British Medical Journal *in 2006 [9] called for more research into 'nuances in other types of study design' (i.e. non-trial studies) and other authors have recommended that more empirical work be carried out into the impact on participants of receiving research results [2,5,10,11]. We sought to explore the possible benefits and harms of the feedback given to participants in the longitudinal 'West of Scotland Twenty-07 Study'.

## Methods

We conducted in-depth interviews with men and women who had participated in the fifth and most recent wave of data collection for the 'West of Scotland Twenty-07 Study: Health in the Community', a 20-year longitudinal study based in the Greater Glasgow area, UK [12]. The Twenty-07 Study began in 1987 and has followed three age cohorts born 20 years apart (in the early 1970s, 1950s and 1930s). Detail on the study can be found elsewhere [12], but briefly the main waves of data collection have included an extensive face-to-face interview by a trained nurse interviewer that covered: self-reported health and health care use; a number of mental health scales; family, social and economic circumstances; behaviours; life events; opinions; and an intelligence test. In addition, a number of physical measures have been taken including: height; weight; waist and hip circumferences; lung function; blood pressure; pulse; and reaction times. Blood samples were added to the suite of physical measures in wave 5, which was carried out between September 2007 and October 2008.

Twenty-07 Study participants have always received immediate feedback of their blood pressure from the nurse interviewer at the time of their interview. In addition, all participants have received periodic aggregate Study results in the form of a newsletter. During wave 5, participants were given the option of receiving their individual physical measures (height, weight, BMI, body fat percentage (measured using Bioelectrical Impedance)) and blood results (total and HDL cholesterol, and their ratio, and glycated haemoglobin A_1c _(as a percentage)) in a feedback letter. Of the 95% (n = 2, 425) of all wave 5 Twenty-07 Study participants who requested a feedback letter, most received it around three months after their interview. Nearly all of those people who chose not to receive feedback did not provide a blood sample. They were slightly more likely to be female, from the younger cohort and from manual social classes compared with the 95% who wished to receive their feedback letter; they also were more likely to be overweight and have high levels of body fat. Blood results were more likely to be elevated among the few in this group who had any blood results.

The weight-related and blood measures given in the letters were put into context for participants; for example, the words 'underweight', 'normal' and 'overweight' were used in relation to the BMI result; for body fat the normal range for men and women was provided; and for the two blood results respondents were recommended to see their general practitioner (GP) if their results were over levels set by the Study's clinical advisors and agreed by the ethics committee (see Table 1). The Twenty-07 Study set the BMI cut-offs for the feedback letter at <19 for underweight, 19-27 for normal and ≥ 27 for overweight, on advice from Twenty-07 Study Clinical Advisers. However, although in the feedback letter 'normal' BMI was defined as having a BMI of less than 27, for this study we used the World Health Organization (WHO) definition with an upper limit 25. We therefore did not include those respondents with a BMI of 25 to 27 in the overweight group as this is not what the letter told the respondents. The overweight group therefore started at a BMI of 27.

**Table 1:** Sentences given in the feedback letters

	Sentence in feedback letter
**BMI **(kg/m^2)^	
<19	This suggests that you might be underweight.
19-26.9	This suggests that your weight is probably normal
≥27	This suggests that you might be overweight.

**Body composition All letters**	Studies have suggested that the recommended percentage of body fat is around 20-31% for women and 13-21% for men.

**Cholesterol (ratio of total to HDL cholesterol)**	
≤4	This is within the normal range.
>4 (no statin reported)	This is a little high and you may wish to consult your GP about the results.
>4 (statin reported)	This is a little high but we note that you reported taking statins and we assume you and your GP are monitoring this.

**HbA**_**1c**_	
<6%	This is within the normal range.
≥6% (no diabetes reported)	This seems to be a little high and we recommend that you have your blood glucose level checked by your general practitioner or practice nurse. You should not eat or drink before you undertake this test, please ask your practice for advice on this when you make your appointment.
≥6% (diabetes reported)	This seems to be a little high but we note that you are aware of your diabetes and assume that you and your GP are monitoring this.

This qualitative study involved in-depth interviews (both face-to-face and telephone) with participants who had received a feedback letter from the Twenty-07 Study in the previous six months. Ethics approval was granted from the University of Glasgow's Faculty of Law, Business and Social Sciences Ethics Committee.

We sought to recruit equal numbers of: men and women; three BMI groups (normal, overweight and obese); and, two age cohorts (1970s and 1950s). We also sought to conduct equal numbers of face-to-face and telephone interviews to explore any potential respondent bias that might have been associated with the visible weight status of the two researchers (both with normal BMI, and both female aged in their 30s and 40s). Forty-eight interviews were planned, but two extra interviews with overweight men were carried out to include men with a BMI >27 *and *high body fat, as two of the orignal interviewees sampled in this group had a BMI >27 but low body fat.

A total of 535 participants (from 2,604 in wave 5) were eligible to participate in this qualitative follow-up study, based on the following eligibility criteria: they had received a feedback letter in the previous six months; they were from the 1950s or 1970s cohorts; they had not been excluded from any measure based on the fieldwork protocol (e.g. pregnant women); and, they had received a standard feedback letter (rather than one with an additional sentence inserted at the request of our clinical advisers to convey an additional result from the blood test results, for example, vitamin deficiency). Estimating a 1 in 4 response to mailed invitations, based on previous Twenty-07 qualitative sub-sample studies, we sent a total of 377 invitations (randomly selected from those eligible); the response was 21.2% (n = 80), with variation by age and BMI. Of these, 50 interviews were carried out (the remaining 30 were not required as we had interviewed to our quota within each target group). Most face-to-face interviews were carried out in the participant's home (the two exceptions were conducted in University settings); all, except one, telephone interviews were carried out with the participant at home (one participant was on a mobile phone travelling as a car passenger during the interview).

This study was part of a collaborative project funded by Cancer Research UK (CRUK), which aimed to investigate response to being informed of weight status and to different issues around weight-related terminology - data on the latter will be reported elsewhere. The interview questions pertaining to expectations of and response to feedback are given in Table 2. Interviews were audio-recorded (with consent obtained in writing for face-to-face interviews and audio-recorded for telephone interviews) and transcribed verbatim. Transcripts were analysed using the Framework Approach, where data are coded, indexed and charted systematically, then organised using a matrix or framework [13]. The matrix contained a row for each participant and a column for each theme. A benefit of this approach is that it is very transparent and allows interpretations to be viewed and assessed by others. Framework analysis begins deductively from the study aims and objectives, with results being grounded (in accounts) and inductive [14]. KL, CG and MB read all transcripts; KL conducted the early coding, which was discussed with two other researchers (CG and MB) as a form of double coding. Constant comparison was carried out to check for deviant cases as well as similarities, in an iterative process. QSR NVivo8 software was used to assist the systematic data analysis and organisation. During analysis, we explored participants' attributes (e.g. gender, age), in which we had an *a priori *interest, against the various themes to rigorously explore emergent patterns in response by age and gender.

**Table 2:** Topic guide (selected questions that investigated participants' experiences of receiving individualised feedback following their wave 5 Twenty-07 interview)

Did you have any expectations of what your results might have been? (Probe, explore what they were)
When you got your letter through, and you read over it, what was your initial reaction to your results? (Explore interpretation of each result)

Did you discuss your results with anyone? (Probe who, what was said...)

Did your results make you think differently about yourself? In what way?

Have you taken any action (done anything) in response to your results? What and why?

What are your views towards taking part in the Twenty-07 Study?

What, in general, did you think about being offered the feedback after your last interview?

In the next section, quotations will be followed with the type of interview, participants' key attributes and their physical measures (BMI and body fat) and blood results (ratio of cholesterol and whether the diabetes test was normal or high) in the language fed back to them in the letter - e.g. Tel 2, Female, age 55, overweight, BF normal, chol high, HbA_1c _normal. Abbreviations will be used: BF for body fat and chol for ratio of cholesterol.

## Results

Table 3 shows how the 50 interviewees compare to all Twenty-07 Study participants. The majority of the 50 participants had participated in all 5 waves (78.0%, compared with 69.8% of all Twenty-07 Study participants) and were mostly from professional households (58.0%, compared with 49.0% of all wave 5 participants), although none of these differences were statistically significant at the 95% level.

**Table 3:** Characteristics of participants in current study and wave 5 of Twenty-07 Study

	Male	Female	All sub-study participants n = 50 (%)	All other wave 5 Twenty-07 participants (1970s and 1950s cohorts) n = 1864 (%)		P value for chi-square
**BMI category**			(% ) Range*	(% ) Range*	**N**	**0.002**

**Normal **(19.0 to 24.9)	8	8	(32.0) 19.9 - 24.0	(26.6) 19.1 -25.0	492	

**Overweight **(27.0 to 29.9)	10	8	(36. 0) 27.1 - 29.8	(22.2) 27.0-30.0	413	

**Obese **(30.0 +)	8	8	(32.0) 30.1 - 44.6	(29.6) 30.0-63.4	558	

***Not eligible †***	-	-	-	(21.5)	401	

**% Body Fat**						0.315

**Within quoted ranges (see Table 1)**	9	5	(28.6) Men 16.0-20.4 Women 27.2-30.9	(24.1) Men 13.0-21.0 Women20.2-30.9	449	

**Above quoted ranges**	16	19	(71.4) Men21.6-33.0 Women 31.1-53.1	(68.5) Men 21.1 -50.9 Women 31.1-61.7	1276	

***Not eligible ¥***	1	-	-	(7.4)	139	

**Cholesterol**						0.061

**Normal **(≤4 mmol/L)	11	17	(57.1) 2.3 - 3.9	(47.5) 1.80-4.0	886	

**High **(>4 mmol/L)	14	7	(42.9) 4.1 - 7.9	(39.2) 4.1-12.3	731	

***Missing***	1			(13.3)	247	

**HbA1c**						0.060

**Normal **(<6%)	24	22	(93.9) 4.3 - 6.0	(81.3) 3.4-6.0	1516	

**High **(≥ 6%)	1	2	(6.1) 6.1 - 9.5	(5.2) 6.1-13.6	96	

***Missing***	1			(13.5)	252	

**Registrar General's Social Class for head of household**			**Percentage**	**Percentage**		0.585

**Professional/managerial household **(I&II)	16	13	58.0	49.0	913	

**Skilled workers **(IIIn-m and man)	9	7	32.0	36.5	681	

**Semi or unskilled household **(IV&V)	1	4	10.0	13.8	257	

***Missing***	-	-	-	0.7	13	

**Wave participation**			**Percentage**	**Percentage**		0.125

**Taken part in all 5 waves**	21	18	78.0	69.8	1314	

**Missed 1 wave**	1	2	6.0	17.0	311	

**Missed more than 1 wave**	4	4	16.0	13.1	239	

* Numbers rounded to 1d.p.

† Those with missing data, underweight or overweight with BMI between 25 and 27.

¥ Those with missing data or body fat less than 'normal range'.

### Expectations of results

Before discussing their response to the results in their feedback letter, participants were invited to reflect on any expectations they had about their letter. A range of expectations were discussed. One group of participants did not give the letter any thought in the period between the main study interview and receiving the letter, or impassively described their expectations.

I had no concerns or anything, so I was quite, yeah, I'd kind of forgotten about it, to be honest, until it came through. (Int 14, Female, age 35, normal weight, BF normal, chol normal, HbA_1__c_ normal)

Around one in three participants offered such a description of their expectations, with slightly more older participants and women expressing these expectations.

A second group of participants were not anxious, but actually quite positive when describing their expectations. This group tended to report being quite healthy and health conscious (e.g. to describe eating healthily and watching their weight) or report having a health condition and be in regular attendance at their GP or other health professional, thus making them aware of their health status in relation to weight and/or cholesterol:

No, I wasn't worried at all, I was looking forward to it... I know a lot about food, I've been a vegetarian since I was [young age], so I'm quite good at it... I know how to read a food package, I know what's in things and to me that's common knowledge; I can't eat something without investigating it first. (Int 24, Female, age 35, normal weight, BF normal, chol normal, HbA_1c _normal)

I didn't expect any great shocks, because I'm one of these people who have over the years had a great deal of medical attention one way or the other through the NHS, and probably I was well monitored. (Int 22, Male, age 55, normal weight, BF normal, chol normal, HbA_1c _high)

Some men in this group considered that the physical measures and blood test had been like having a 'free medical' (Int 22, Male, age 55, normal weight, BF normal, chol normal, HbA_1c _high), which they welcomed.

A third group, around a quarter of all participants, revealed slight concerns or anxieties about receiving high results, either in general terms 'I had a vague idea what was going to be in it [letter], that it was probably not going to be good.' (Int 11, Female, age 35, overweight, BF high, chol normal, HbA_1c _normal), or specifically about either the physical measures or the blood results:

I was a wee bit concerned that my cholesterol might be high... I was a wee bit concerned I was getting quite diabetic because again my sugar level was quite high so that was a slight concern.(Tel 20, Male, age 55, overweight, BF high, chol high, HbA_1c _normal).

Five of these participants used the word 'concerned' about their impending results and were tentatively expecting negative results ('think' they will be bad); others were more definite in their expectations of negative results. Women more often than men spoke negatively about their expectations. There were no differences by age.

More participants described not knowing what to expect from their blood results than from their weight-related results. Despite some reported anxieties about blood results, what emerged clearly in participants' narratives of their negative expectations was the greater emphasis in discussions of their weight results, even among those who reported never having had their cholesterol tested before. The few participants who were apprehensive about their blood results also spoke of concern about their weight-related results.

### Initial response to results

Participants' response to results did not always match with their prior expectations. Overall, half of participants' descriptions of their initial response were not in line with their expectations: some received better feedback than expected whereas some received worse.

Fifteen participants received results which were either better than expected or confirmed their positive expectations, so their result either made no difference to them or caused them to feel 'happy', 'glad' or 'pleased'. One man who 'didn't expect any great shocks' later described his initial response:

I was really pleased that all the indications were that I was absolutely where I should be for my age and height and I was really quite gratified about all that. (Int 22, Male, age 55, normal weight, BF normal, chol normal, HbA_1c_ high)

The complexity of narratives meant that some people who were overweight or had a high total to HDL cholesterol ratio were also pleased with their feedback results. These participants often placed their results in the context of prior 'worse' health, such as having a ratio of cholesterol of 7 mmol/l in the past and receiving a result of 5 mmol/l in their feedback letter (even though this had been fed back in their letter as 'high' with a recommendation to visit their doctor).

I felt they were actually quite good; I was quite happy with them... the [blood] result wasn't as bad as I thought it was going to be ... overall I was happy with the results. (Tel 20, Male, age 55, overweight, BF high, chol high, HbA_1c_ normal)

In contrast, ten participants who expressed neutral or positive prior expectations went on to describe a negative initial response to their results. One woman described being unconcerned in advance about receiving her results as she believed herself to be 'quite healthy so I wasn't really concerned'; however, she was taken aback by her cholesterol and BMI results:

I was quite surprised when I did get it [letter] through... I felt that my cholesterol was high... And that I was over, well I knew I was overweight but I think I was actually, I'm actually classed as obese [respondent checked result on Internet]. (Tel 21, Female, age 35, obese, BF high, chol high, HbA_1c_ normal).

Eight of these participants, almost all from the younger age group, referred to the impact of seeing their results in 'black and white'. Seeing their results in the letter felt stark, making them feel unable to ignore their overweight or high cholesterol status.

I suppose seeing it in black and white, you know, where you actually think, 'Oh that is what that says' and 'God, I am fat!' (Int 12, Female, age 35, overweight, BF high, chol normal, HbA_1c_ normal).

For two participants, seeing their results in the letter confirmed their positive expectations of 'normal' results or offered no surprise to see a high result:

I've always known that my weight is classed as overweight so it didn't really surprise me when I saw it in black and white. (Int 2, Male, age 35, overweight, BF normal, chol high, HbA_1c_ normal).

### Emotional response

Several participants revealed a strong negative emotional response to their results. Women, in particular, often used strong language to describe their initial response, including being 'shocked', 'concerned' and 'surprised'. One 55 year old woman (Int 9, Female, age 55, obese, BF high, chol normal, HbA_1c _normal) cried when describing her weight-related results.

Within these emotional responses, participants were frequently candid in their summation of their initial reaction to their results:

Every bit of information in there (apart from height), em BMI, the body fat composition, was just dreadful. Half of me is fat. More than half of me probably. Can't remember exactly, but half of you is fat. You know, and I think, 'No. I've got all these bones as well, and I've got lungs and I've ... No no, the majority of you is fat', you know. And you think [sighs], 'No. No. This is just dreadful. I don't want to be a big ball of fat'. (Int 6, Female, age 35, obese, BF high, chol high, HbA_1c _normal).

This forthrightness was more often expressed in relation to the weight-related results than the blood results, even among participants who had received a high cholesterol result. One 35 year old woman received both a BMI and a cholesterol result that were just into an 'unhealthy' category, but during her interview she focused on being concerned about her weight result:

...To think that you're just young and you're healthy and you're just carrying too much weight is quite... aye, I was quite concerned about it actually. (Tel 21, Female, age 35, obese, BF high, chol high, HbA_1c _normal)

However, a few participants made light of their negative results.

Int: So seeing your weight result, how did you feel about that?

Int 15: Well I know what the answer is there: I know I could do better. It's obvious, isn't it? [2 secs] Amputation's all that's left to me [laughs]. Maybe I could get by without my head [laughing]. How much does a head weigh?

(Int 15, Male, age 55, obese, BF high, chol high, HbA_1c _normal)

### Behavioural response

Several participants reported altering their lifestyles after receiving their letter, citing it as motivating their behaviour change:

I think I was expecting it to be a little bit better. But you know, I kind of eh, sometimes you can kid yourself on a wee bit. So you think you're doing better than you actually are. So it's motivated me now a bit more than I had done in the past, so I'm doing a lot more exercise and eating a lot more healthier now.

[Sic] (Tel 7, Male, age 35, overweight, BF normal, chol normal, HbA_1c _normal)

One woman reported that she had lost four stones (~25 kilograms) since receiving her feedback letter; one man reported losing over a stone (~6 kilograms). Most (9/10) of those who reported behaviour change were in the younger age group. However, more participants (15/50) spoke about being motivated to make changes to their diet and/or exercise as a result of receiving the feedback, but admitted they had yet to implement any change. Twelve of these participants were in the older age group.

...as far as the BMI, etcetera, was concerned I wasn't surprised because I know that I'm overweight, I know that I should try and lose weight but, as I say, I don't get obsessive about it because it just gets me depressed. (Tel 22, Female, age 55, obese, BF high, chol high, HbA_1c _normal).

Thus, it seems the 'stark' reality of seeing their results in the letter seemed to resonate more with the younger participants who subsequently appeared more likely to take steps to change their health behaviour in comparison with the older participants.

It is also important to note that many participants reported no change in behaviour as they had received all 'normal' results and believed they were maintaining their health so no modifications were required.

### Understanding their results

Participants revealed a range of understandings of their individual weight and blood results. The majority had a general understanding of these results. Most understood the BMI measure was a weight-related measure, but whilst relating it to weight they often used words which revealed their uncertainty:

I'm not really terribly sure exactly what it [BMI] means. Does it mean that you've got too much fat you know per whatever in proportion to what your frame is, what your height is? (Int 17, Female, age 55, normal weight, BF normal, chol normal, HbA_1c _normal).

Cholesterol results were generally perceived by participants to be related to diet and that the lower the better:

I'm not a really healthy eater, but I wouldn't have thought that I ate such a high fat content that would push my cholesterol up. I don't smoke, my drink is... I drink moderately, so it was a wee bit surprising, yes. (Tel 5, Male, age 35, overweight, BF normal, chol high, HbA_1c _normal).

Although most could not recall their actual weight and blood results in terms of the specific number, they could recall whether their weight and blood results were 'normal' or 'high'.

Whilst many were unable to explain the BMI measure beyond it being a weight-related measure, it nevertheless resonated with many participants, whether normal, overweight or obese. Other results were discussed less by participants. The body fat measure, in particular, was rarely mentioned in any detail by participants, who favoured discussing their BMI and ratio of cholesterol. Even when prompted, participants often could not remember their body fat result or failed to understand the relevance of the percentage given to them, despite being told of the 'normal' range in their letters.

I don't know, I don't remember that result, if it was 33% I wouldn't have any context, I don't know how good, bad or indifferent that is.

(Tel 9, Male, age 55, obese, BF high, chol high, HbA_1c _normal)

Whilst understanding of the measures was often discussed in general terms, a few participants reported more detailed knowledge, such as for the BMI cut-off points:

Well above 25 you start to sweat and above 30 you start to worry. (Tel 9, Male, age 55, obese, BF high, chol high, HbA_1c _normal).

During discussions of their understanding, some participants queried the validity of their result(s), in particular the BMI measure: eight men (BMI range 27.6 - 36.6) rejected the BMI on the grounds that the measure does not take into account fitness or muscle - features all of these men believed they had. Four of the men had a BMI in the overweight range but had normal body fat measures; however, three of these men had a BMI in the obese range, had elevated body fat and ratio of cholesterol results which ranged from 4.8 to 7.2. One man who was obese with a high body fat (30.6%) and high cholesterol (4.8) reported:

I'm overweight but I'm not obese, I don't think, although technically I might be in the BMI thing, which is, of course, tosh...if the BMI guy who wrote the chart wants to race me, I will! (Int 15, Male, age 55, obese, BF high, chol high, HbA_1c _normal)

## Discussion

Despite the ethical imperative to offer research participants the choice of receiving study results, there remains a striking lack of empirical research on how people respond to individualised feedback, with the majority of the literature focusing on feeding back aggregate study data or, in the case of individualised results, to participants of genetics research [4,6]. Our study fed back individual data to participants in a community sample; as such, our examination of study participants' expectations and subsequent response to individual feedback adds new and important knowledge to this field.

Our results reveal minimal harm done to some respondents. We found no simple relationship between feeding back a 'negative' result and having a corresponding negative response or positive behaviour change. Instead, a complex picture was found, which depended on factors such as age, gender, expectations about results and, drawing on the health education literature, how the feedback in some cases interacted with individuals in different stages of health behaviour change (precontemplation, contemplation, preparation, action and maintenance) [15]: for example, one woman understood she was obese and had contemplated weight loss but upon seeing her results in 'black and white' subsequently took action and reported significant weight loss.

We found a variety of understandings about cholesterol, diabetes and BMI, which is similar to other work [16]. Nevertheless, most participants had a good general understanding of the four results fed back to them (knowing the lower the better), so it is possible that understanding of the results among our follow-up study sample was sufficient but that respondents were engaging in recall only of perceived self-relevant information, which is similar to other work which fed back cholesterol information to participants [17].

### Limitations

The findings must be considered in the context of various potential biases: the participants were drawn from the fifth wave of a longitudinal study and constitute a potentially highly study-loyal sample - all 50 participants have remained with the Twenty-07 study over 20 years. The views of those who 'drop-out' could differ from those of loyal participants - this could have implications for when and how feedback is offered as part of longitudinal studies. Participants from higher social class households were overrepresented in the current follow-up study, although this broadly mirrors the main Twenty-07 Study sample. Nevertheless, 21 of the 50 participants were from households in which the head of household had a semi or unskilled occupation and no differences in views between the two groups were detected in the analysis. It is also possible that social desirability bias could have been introduced, with some participants possibly being less candid than others in their re-telling of their response to their feedback. The use of telephone interviews along with face-to-face interviews allowed us to explore any potential respondent bias that might have been associated with the visible weight status of the researchers; none was apparent in analyses. The attention paid to the feedback on weight as compared with other measures (e.g. blood results and % body fat) suggests that our findings may not be generalisable to individualised feedback of other measures. More research is needed to investigate this. Given the qualitative nature of the follow-up study, and hence the small numbers, we do not know what proportion of all Twenty-07 Study participants who received a feedback letter would have implemented behaviour change in light of their feedback results. Thus, the effect of feedback on the longer-term validity of longitudinal studies is not immediately obvious from these data. The low response rate reveals it was difficult to recruit these longitudinal study participants to the sub-sample study, which is perhaps a product of only having recently exposed participants to a lengthy main study interview (which lasted an average 2 hours and 43 minutes [18]) and perceptions that their 20-year participation had come to an end. Participants were not asked if they would choose to receive the feedback letter again, which would have assisted in contextualising potential harms caused.

### Implications for future research

We assumed the four results fed back would be clearly known and understood by participants, being fairly common measures and the fact the letter they received contained their results against population standard ranges. However, evidence from this study suggests this may not be the case and it may be sensible to provide some additional information about the results fed back, for example by providing a supplementary leaflet which explains the measures. Further research should assess the impact of providing additional information on understandings and hence the potential enhancement of the benefit of individualised feedback to participants.

Perhaps counter intuitively, participants described the weight results having more impact than the blood results. Perhaps this reflects the easier 'visibility' of such risk factors for subsequent ill-health, rendering them more available for discussions in day to day discussions of the implications for future ill-health [19]. Researchers should tread cautiously in assuming which results could cause upset in light of sparse empirical evidence on feeding back individualised data to participants. The results of this study suggest researchers may wish to avoid assuming participants will be less affected emotionally to the receipt of visible results such as weight than invisible results (e.g. cholesterol). Careful wording of feedback letters may therefore be important to assuage negative impact, for all results whether visible or invisible. Thus, we concur with Dixon-Woods et al [5] who call for further evidence on best practice to avoid 'assuming that providing research results to participants is straightforward'. This underscores the importance of continuing to monitor the effects of different types of individualised feedback data on participants. For example, researchers involved in randomised control trials may grapple with conveying complex genetic data to participants in meaningful way (although not all genetics-based studies need be trials). However, given the ethical imperative to offer results to study participants, and the potentially high percentage who will wish to receive it [4], researchers are increasingly required to provide clear feedback which will cause minimal harm to participants, and should consider strategies for giving additional support, guidance or information to participants following feedback to help them to interpret their results.

The existing literature has raised questions about how best to feed back individualised data within different types of studies. Although this follow-up study is moderate in size these ethical issues are potentially transferable to other community-based studies which are feeding back data to participants, for example the Scottish Health Survey, Whitehall studies and the MRC-funded Caerphilly Prospective Study (CAPS) - UK Biobank also provide participants with individualised results such as blood pressure and BMI [20]. Thus, a not insignificant number of people are currently receiving (or potentially will receive) such feedback, so the process of refinement deserves attention.

Jeffery et al. [21] outlined ethical dilemmas in feeding back data to participants in a longitudinal study, and the implications to validity of longitudinal studies. Their short article offered no conclusion; our study reveals care is needed in how individualised data are fed back, as the age and gender differences in response to our feedback suggest potential non-uniform changes in subsequent observed data. It would seem that the feedback did, for some, act as an unintended intervention. This concurs with Dixon-Woods' assertion that providing research results 'constitutes an intervention in its own right' [5]. Thus, the non-interventionist nature of longitudinal studies could be compromised. There may be nothing that researchers can do about this except monitor potential biases and then report them appropriately in subsequent publications which report subsequent analyses of the research data. We would recommend that researchers on longitudinal studies, who provide results which could change behaviours, perhaps follow-up a sub-sample to assess the impact feedback may be having on behaviours so that potential biases are monitored and analyses and dissemination are conducted and reported accordingly. In addition, it is important to monitor subsequent participation of different feedback to inform later waves, as any attrition due to feedback could affect the response rates and, subsequently, bias.

## Conclusions

There remains insufficient evidence in the literature to determine how best to conduct individualised feedback as part of non-RCT research to enhance benefits and reduce harms; in the case of longitudinal studies, it remains unclear how to reduce potential bias, unintended interventionist effects or impact on subsequent wave participation. Such effects may vary by study type as well as by the type of data fed back to respondents.

## Authors' contributions

All authors contributed to the development of the ideas in the manuscript and read and approved the manuscript. KL wrote the first draft and collated comments from authors for subsequent iterations. All authors reviewed drafts of the manuscript, and provided feedback.

## Competing interests

The authors declare that they have no competing interests.

## Pre-publication history

The pre-publication history for this paper can be accessed here:

http://www.biomedcentral.com/1471-2288/11/10/prepub
